# Neural Correlates of Group Versus Individual Problem Solving Revealed by fMRI

**DOI:** 10.3389/fnhum.2020.00290

**Published:** 2020-08-28

**Authors:** Ilya Yu. Shpurov, Roza M. Vlasova, Alena D. Rumshiskaya, Renata I. Rozovskaya, Elena A. Mershina, Valentin E. Sinitsyn, Ekaterina V. Pechenkova

**Affiliations:** ^1^Research Institute of Neuropsychology of Speech and Writing, Moscow, Russia; ^2^Department of Psychiatry, The University of North Carolina at Chapel Hill, Chapel Hill, NC, United States; ^3^Davydovsky City Clinical Hospital, Moscow, Russia; ^4^Radiology Department, Federal Center of Treatment and Rehabilitation, Moscow, Russia; ^5^Department of Psychiatry, University of Pittsburgh, Pittsburgh, PA, United States; ^6^Medical Research and Education Center, Lomonosov Moscow State University, Moscow, Russia; ^7^Laboratory for Cognitive Research, National Research University Higher School of Economics, Moscow, Russia

**Keywords:** cooperative interaction, problem solving, group problem solving, second-person neuroscience, social brain, default mode network, fMRI

## Abstract

Group problem solving is a prototypical complex collective intellectual activity. Psychological research provides compelling evidence that problem solving in groups is both qualitatively and quantitatively different from doing so alone. However, the question of whether individual and collective problem solving involve the same neural substrate has not yet been addressed, mainly due to methodological limitations. In the current study, functional magnetic resonance imaging was performed to compare brain activation when participants solved Raven-like matrix problems in a small group and individually. In the group condition, the participant in the scanner was able to discuss the problem with other team members using a special communication device. In the individual condition, the participant was required to think aloud while solving the problem in the silent presence of the other team members. Greater activation was found in several brain regions during group problem solving, including the medial prefrontal cortex; lateral parietal, cingulate, precuneus and retrosplenial cortices; frontal and temporal poles. These areas have been identified as potential components of the so-called “social brain” on the basis of research using offline judgments of material related to socializing. Therefore, this study demonstrated the actual involvement of these regions in real-time social interactions, such as group problem solving. However, further connectivity analysis revealed that the social brain components are co-activated, but do not increase their coupling during cooperation as would be suggested for a holistic network. We suggest that the social mode of the brain may be described instead as a re-configuration of connectivity between basic networks, and we found decreased connectivity between the language and salience networks in the group compared to the individual condition. A control experiment showed that the findings from the main experiment cannot be entirely accounted for by discourse comprehension. Thus, the study demonstrates affordances provided by the presented new technique for neuroimaging the “group mind,” implementing the single-brain version of the second-person neuroscience approach.

## Introduction

The idea that a group is more than the sum of its members is one of the basic principles of social psychology. People feel and behave differently when doing things together, as if their individual minds become components of a “group mind.” A large body of empirical evidence supporting this metaphor comes from the literature on cooperative social interactions, such as group problem solving. For example, the performance of a small group in problem solving may differ from the aggregated individual performances of all group members or the most productive group member working alone (Lorge et al., 1958; Hill, 1982; Laughlin et al., 2006). Groups may systematically outperform or underperform any of these baselines (Kerr and Tindale, 2004) demonstrating group gain (“group superiority effect”) or group loss, respectively. A seminal study by Woolley et al. (2010) suggested that small groups may be treated as collective subjects with their own level of collective intelligence, and that this level does not correlate with either the average or the best individual intelligence of the group members, but may be predicted by gender, average social sensitivity, and in-group discussion style. As the replicability of this phenomenon is still debated (Bates and Gupta, 2017; Credé and Howardson, 2017) the question arises of what conditions are necessary for the collective intelligence to emerge, and whether it can be revealed in computer-directed communication or via face-to-face interaction only (Engel, 2015; Barlow and Dennis, 2016).

Human cooperative social interactions involve communication and mutual control of behavior, requiring many specific cognitive functions and abilities, such as theory of mind (Premack and Woodruff, 1978) joint attention (Baron-Cohen, 1991) shared conceptual common ground (Tomasello, 2008) and the ability to set and achieve a joint goal (Leontiev, 1978). When people cooperate, they practice the off-loading and outsourcing of cognition (Hari and Kujala, 2009). Therefore, human behavior and mental processes are not only quantitatively but also qualitatively different when a group rather than an individual reasons through a problem. But what happens differently in the brain?

No straightforward answer to this question is known, as social neuroscience is tightly constrained by theoretical and methodological limitations, labeled by Northoff and Heinzel (2006) and by Schilbach et al. (2013) as “third-person neuroscience.” In creative writing, the first-person point of view implies that the narrator is a character in their own story, while a third-person point of view narrator represents a spectator who is not interacting with the story characters. Following this metaphor, the model of a participant in a neuroscientific experiment has long been similar to a reader of a third-person point of view novel who may observe others and infer their thoughts, but does not interact with them. Most neuroimaging data about the so-called social brain – the neural substrate of human communication, social cognition, and interaction (Brothers, 1990; Frith, 2007) – are acquired while a participant responds to social stimuli that can be implemented successfully in a laboratory setting (e.g., faces, biological motion, utterances, descriptions or depictions of the social behavior of other humans). Paradigms of this kind enable a researcher to control for experimental variables and artifacts generated by overt speech or head motion. However, there is a potential difference in the brain activity of a participant who remains a passive spectator of other people’s social interactions and a person who is actively engaged in group dynamics (Hari et al., 2015). Following the creative writing metaphor, we may say that the new model of the participant would be similar to the second-person point of view in fiction when the narrator addresses the readers as “you” and immerses them into the story. Therefore, there is a call for the interactive “second-person neuroscience” (Schilbach et al., 2013) which is capable of uncovering neural mechanisms different from those involved in social observation (Redcay and Schilbach, 2019).

Multiple attempts to increase ecological validity in research on the social brain have involved all of the main neuroimaging modalities (Babiloni and Astolfi, 2014). Here, we will mainly discuss fMRI techniques for studying online (i.e., feedback-contingent) interactions. The most popular method is a computer game in which a participant in the scanner believes he or she is playing remotely with invisible counterparts in the control room. A game may be replaced by a chat (Warnell et al., 2018). Most often, this technique involves subject deception and the simulation of real interactions, with all other participants being mimicked by the experimenter or a computer in a way that allows the manipulation of experimental variables (but see McCabe et al., 2001; Rilling et al., 2002; Chaminade et al., 2012 for exceptions). Assistants involved in the experiment take part in the practice sessions together with real participants, and their photographs, names, or even short videos are presented on the screen in the scanner to support the cover story and make the participants believe that they interact with a real human player. This technique has often been used in game theory tasks (Rilling et al., 2004; Fukui et al., 2006; Kircher, 2009; Hollmann et al., 2011; Güroğlu et al., 2014; see also Gallagher et al., 2002 for an early PET study), but has also been applied to pattern construction games (Decety et al., 2004) and conformity studies (Berns et al., 2005).

A second technique is a video chat between someone in the control room and the participant in the scanner. Unlike the simulation method, video chat enables remote but face-to-face online communication (Redcay et al., 2010). The virtual character technique introduces a realistically human virtual character that communicates with the participant in a face-to-face manner and systematically implements experimental conditions, such as gaze direction (Schilbach et al., 2006, 2013) or the demonstration of pain from an electric shock in the virtual Milgram’s paradigm (Cheetham et al., 2009).

Hari et al. (2015) proposed that the key to the neuroimaging of real social interactions lies in the advanced technology of hyperscanning, which provides the simultaneous registration of brain activity in two or more interacting participants who communicate either face-to-face or via the Internet (Montague et al., 2002; Koike et al., 2015; Czeszumski et al., 2020). During fMRI, the dual-head coil allows for hyperscanning while participants are involved in simple face-to-face sensorimotor interactions (Lee et al., 2012). Paradigms for the Internet-mediated approach include game theory tasks (Tomlin et al., 2006) and real-time video joint attention tasks (Saito et al., 2010; Bilek, 2015; Koike, 2016; Goelman et al., 2019). With other imaging modalities that are less sensitive to the experimental environment and participant motion, such as EEG and fNIRS, there have been several successful attempts to study more complex interactions, such as gesture communication (Dumas et al., 2010) playing a card game (Astolfi et al., 2010) duet guitar performance (Müller et al., 2013) cooperative singing (Osaka et al., 2015) and even live patient surgery by an operating room team (Stevens et al., 2019). Redcay and Schilbach (2019) classify this method as the simultaneous dual-brain approach to second-person neuroscience.

The main focus of hyperscanning studies is to reveal similarities and covariations in the brain activity of interacting participants (hyperconnectivity) (Burgess, 2013) or to explore more complex systemic neurodynamic features that reveal the presence of team information beyond the level of individual (Stevens et al., 2018). This kind of evidence can be used to test the claim that social interaction generates emergent group-specific properties of brain activity. Such evidence was obtained in an EEG study by Astolfi et al. (2010) who demonstrated that, when two dyads play a card game against each other and share most of the sensory events during the game, the brain activity of the participants synchronizes within, but not across, teams. Similar results were obtained by Koike and colleagues (Koike, 2016; Sadato, 2017) who used fMRI hyperscanning to identify dyad-specific neural synchronization in the right inferior frontal gyrus as a potential neural substrate of joint attention. A series of recent fNIRS hyperscanning studies has also shown increasing inter-brain synchronization in the right temporoparietal junction (TPJ) and the right dorsolateral prefrontal cortex (dlPFC) in cooperating dyads working on creativity problems or playing games (Liu et al., 2016, 2017; Xue et al., 2018).

With the exception of the handful of fMRI studies mentioned above, the hyperconnectivity data obtained thus far do not provide detailed (whole-brain scale, high spatial resolution) information about the location of neural substrates specific to group activity compared to individual activity. In the current study, we propose another method that falls under the single-brain approach in Redcay and Schilbach (2019) classification, but may as well be considered an implementation of the “second-person neuroscience.” It is a complementary alternative to hyperscanning, with a focus on the location of the engaged components of the interacting social brain rather than the fine-grain dynamics. Such an approach requires the direct comparison of neural substrates in group versus individual performance, as revealed by fMRI during online social interactions.

To the best of our knowledge, only two fMRI studies so far have directly compared individual performance and dyad interaction conditions. Decety et al. (2004) utilized a simulation technique making participants believe that on some trials of the spatial pattern game they either cooperate or compete with other players. Sebanz et al. (2007) asked their participants to perform a go-no go task either alone in the presence of the experimenter’s assistant sitting next to the scanner, or in a dyad taking turns with this other person. Both groups reported that the cooperative interaction elicited higher activation in the medial prefrontal cortex (mPFC), which is considered to be a component of the social brain (Kennedy and Adolphs, 2012). Decety et al. (2004) also found activation in the insula as well as in a number of regions that are usually not associated with the social brain, such as the parts of the superior and inferior parietal cortices and superior frontal gyri. This evidence supports the idea that the neural correlates of actual online interactions may be different from the description of the social brain obtained on the basis of an “offline” study of reactions toward isolated social stimuli.

The aim of the current study was 2-fold: to directly address the question of whether the processes of individual and collective problem solving share the same neural substrate, and to investigate the localization of brain activity during real-time social interactions, such as group discussion. As our knowledge about the neural correlates of even classic individual problem solving advanced only recently (Kroger et al., 2002; Goel, 2007; Qiu et al., 2010; Kounios and Beeman, 2014; Bartley et al., 2018) we did not make any predictions about the exact pattern of areas that may be involved in the specific neural implementation of group problem solving. We only expected the engagement of some areas previously described as components of the social brain (Frith and Frith, 2010; Kennedy and Adolphs, 2012; Schilbach et al., 2013) or extra areas revealed by Decety et al. (2004). Additionally, we were interested in whether these areas would show modifications of functional connectivity during group versus individual problem solving, suggesting their operation as a whole large-scale social brain network rather than just a set of co-activated areas.

Data from group and individual problem solving were compared in the same participants. Because pre-existing groups may outperform *ad hoc* groups (Andre et al., 1979; Peter-Wight and Martin, 2011) and in order to keep characteristics of within-group communication and group performance stable throughout the experiment, previously established small groups with substantial experience in collective intellectual activity were selected as study participants. To avoid differential effects of reward, gains and losses were calculated for the whole group and the same payoff matrix was used for both individual and group conditions. Since normal group discussion intrinsically involves conversation, it implies speech production and listening to other voices, and therefore these two aspects were, respectively, controlled. The individual condition involved thinking aloud, which is a technique commonly used in behavioral studies of individual problem solving (Ericsson and Simon, 1993) and a special control condition was introduced to measure brain activation evoked by listening to team members’ scrambled conversations. However, such a control is not exhaustive, because real discussion is still more interesting for people than scrambled utterances and it involves comprehension at sentence and discourse levels. Therefore, we performed an additional control experiment to assess the possible impact of attention toward speech and discourse comprehension on the results of the main experiment.

## Materials and Methods

### Participants

Participants were recruited from among Moscow players of the “What? Where? When?” quiz game. This intellectual sport is very popular in the countries of the former USSR, especially among academics, and is played by teams of six players. Twenty-four teams of three volunteers each were selected so that each pair within each triplet had at least one year of prior experience playing the game together. All participants were between 18 and 45 years of age, native Russian speakers, passed MRI safety screening, reported normal or corrected-to-normal visual acuity and normal hearing, and reported no history of neurological or psychiatric disease. In each team, one right-handed participant who actively took part in the discussion (Player 1; mean age 25 ± 5 years, 10 women) was selected for scanning. All participants provided written informed consent before the experiment. At the end of the procedure, each team was paid according to their performance (mean payment approximately 2,900 Russian rubles). The Interuniversity Ethics Committee of Moscow approved the study.

### Materials and Procedure

Each team completed both a practice session in a classroom and a scanning session on the same day. The entire procedure lasted approximately 4 h. Stimulus sequences programmed using Psychtoolbox-3 (Kleiner et al., 2007) were displayed on a laptop screen during the training session and projected onto a screen in the magnet room during the scanning session. In the latter case, the presentation was automatically synchronized with the imaging sequence using the Current Designs fiber optic response (fORP) interface unit, which was also used for response collection.

**FIGURE 1:**
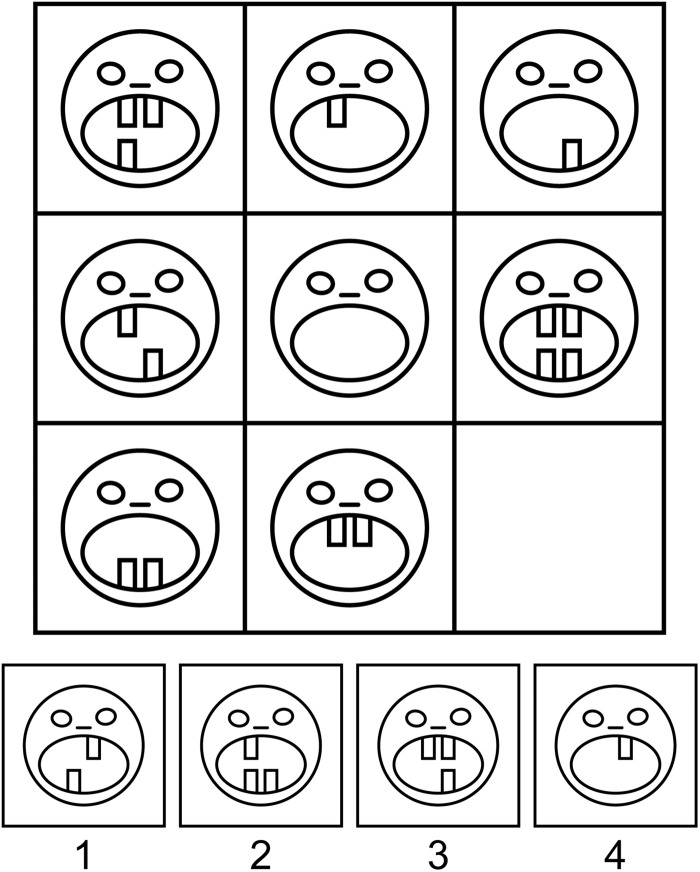
A sample Raven-like matrix problem. Participants have to fill in the lower right corner of the visual matrix with one of the four alternatives by figuring out the principle of the matrix organization. Item created by Maria Falikman.

Participants were administered Raven-like 3 × 3 matrix problems with four response options. Most matrices were adopted from Standard Raven Progressive Matrices, Advanced Raven Progressive Matrices (Raven, 1965, 1976) and the collection of problems designed by A. D. Jensen and the Denmark Mensa Society^1^. Some other matrices were generated by Sandia Generated Matrix Tool (Matzen et al., 2010) or newly constructed. Unlike classic Raven matrices, some items were based on culture-specific encyclopedic knowledge, such as the numeral systems or symbols of different countries. A sample task is shown in Figure 1.

In order to control for task-specific effects, all problems were divided into two sets of 60 items (sets A and B). Half of the participants received set A in the group condition and set B in the individual condition, and vice versa for the other half of participants. Matrices based on similar principles and within the same range of difficulty were equally distributed among A and B. Sets A and B were further split, one half used for the practice session and the other half for the scanning session. Practice and scanning sessions consisted of five runs each. Each run was 12 trials long. The individual and group condition alternated every two trials.

**FIGURE 2:**
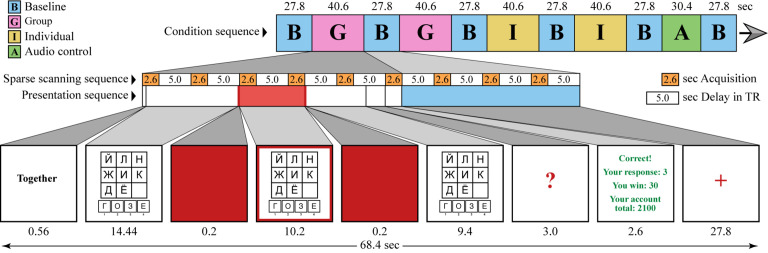
Timeline of the stimuli. Each trial started with instruction indicating whether the subsequent problem should be solved collectively or individually. The subsequent problem solving is split into three time periods: before, during, and after the presentation of the red frame at the edge of the screen. Player 1 is required to keep silent during the red frame period and to speak up during the two other periods. All participants are required to keep silent during the fixation baseline (red cross). Sparse acquisition of the functional MR volumes is illustrated by the white and orange bars.

At the beginning of the practice session, participants were given several examples for a free discussion. Experimenters then selected Player 1 and gave further instructions to the team. For the remaining part of the experiment, each trial started with the briefly shown labels “together” or “alone.” The timeline of the stimuli is shown in Figure 2. In the group condition, all participants discussed the problem, and then Player 1 indicated the chosen response by pressing a button. In the individual condition, Player 1 was required to think aloud while solving the problem alone. The thinking aloud technique was introduced in order to keep the participant’s activity in the individual condition as close to that of the group condition as possible. However, Player 1 tended to produce more utterances in the group conversation than when thinking aloud, which may bias neuroimaging results in favor of the speech areas of the brain rather than the neural correlates of the social interaction *per se*. The following procedure was developed to avoid this issue.

During the practice session, all participants were trained to stop talking when a red cross appeared on the screen (baseline condition). Player 1 was also instructed to keep silent during the presentation of the red frame (from seconds 15 to 25 in each 38-second trial), and then could speak again and reply to other players’ comments until the end of the trial. The duration of the red-frame period was tested in the pilot study and adjusted in a way to keep the full engagement of the participant in the group interaction. With the longer silent period, Player 1 often opted for solving the problem on their own and ignoring their co-players’ conversation. During the optimal silent period, Player 1 reported covert participation in the discussion, i.e., following the arguments of the co-players and internally responding in agreement or disagreement. After the red frame was gone, Player 1 usually immediately joined the group discussion with overt replies. During the scanning session, the red-frame silent period (marked with red in the presentation sequence in Figure 2) provided a period of time in which the participant’s head remained still and made it possible to acquire two functional volumes not corrupted by speech-related head motion artifacts.

Each trial ended with a 3-second period for response (a “?” symbol displayed on the screen), followed by feedback indicating whether the response was correct, the gain or loss from the trial, and the current score. In both the group and individual conditions, the same amount was added to or withdrawn from the whole team’s payment for every correct response or incorrect response, respectively. Rule violations, such as speaking while the red cross was present or avoiding thinking aloud in the individual condition, were treated as incorrect responses.

**FIGURE 3:**
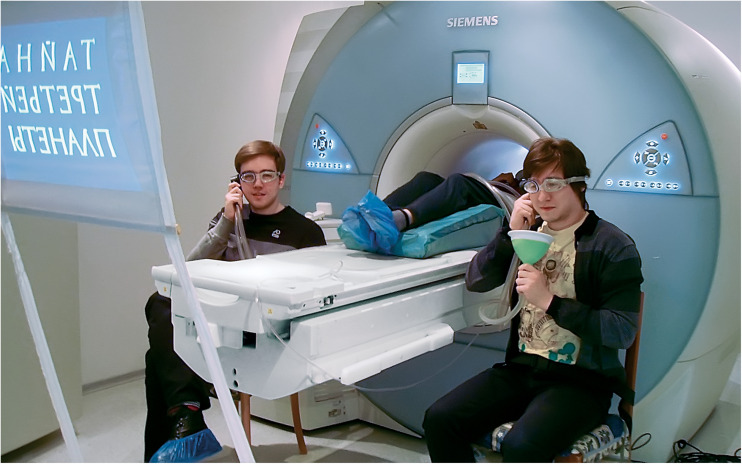
Three members of one of the intellectual sports teams use an MR-compatible communicator system within the setup for the main experiment. The photograph is published with the permission of the participants.

During the scanning session, Player 1 was placed in the magnet bore and the two other players occupied chairs beside the magnet. Extra padding was used to constrain the possible head motion of Player 1. All three participants were interconnected with a custom-made plastic communication system consisting of three headphones and three speaking trumpets. The system substantially reduced interference from the scanner noise, so that participants could discuss problems in the MRI environment as if in a classroom (see Figure 3). Each participant covered one ear with the system’s headphone, which also served as ear protection, and was provided with an ear plug as a protection aid for the second ear. The system was successfully tested in both the 1.5T and 3T Siemens scanner environment with the substantial and stable voice amplification effect observed. Although communication was possible through our system even during the anatomical scans accompanied by the loudest acoustic noise, the best effect was achieved with sparse sampling pulse sequences specifically designed for fMRI studies of speech production and comprehension (Hall et al., 1999; Perrachione and Ghosh, 2013).

The resting condition was implemented as a 27.8-second interval of central fixation (red cross). Another control condition was introduced after every four trials as 30.4-second periods of fixation accompanied by listening to scrambled audio recorded during the practice session and played into the magnet room through loudspeakers. The audio condition was designed to control for the confounding effects associated with the perception of team members’ voices and lexical utterances. As such, it was recorded during the periods when Player 1 was silent. Three-second recording fragments were shuffled in a way that individual words remained mostly preserved but produced non-sense combinations.

### Imaging Parameters

MRI data were collected on a 1.5T Siemens Avanto scanner with a standard matrix head coil. T2^∗^-weighted functional images were acquired using a GE-EPI pulse sequence with TE 50 ms, FA 90°, FOV 230 × 230 mm, matrix size 64 × 64, and voxel size 3.6 × 3.6 × 4 mm. Sparse sampling was applied to reduce scanner noise and improve speech comprehension during the main task. The sparse imaging (delay in TR) technique (Hall et al., 1999) is commonly used in language, hearing, and music studies (Perrachione and Ghosh, 2013). Silent periods (delays), usually several seconds in length, are introduced between the consecutive functional volume acquisitions, preventing contamination of the auditory stimuli presented during these delays by the scanner acoustic noise. In the present study, the effective TR was 7600 ms; one functional volume per effective TR was acquired with a TA of 2600 ms and delay in TR of 5000 ms. Each functional volume covered the whole brain and consisted of 31 slices (4 mm, with a gap of 0.4 mm) oriented parallel to the AC-PC plane and acquired in interleaved mode. A total of 123 volumes were collected in each of the five 16-minute functional runs. Field maps with the same slice prescriptions as the functional images were acquired in the middle of the experiment using a standard Siemens double echo GRE field mapping sequence. T1-weighted MPR structural images were acquired before the functional runs.

### Imaging Data Analysis

Functional data analysis at a single subject level was performed using SPM8 (Wellcome Institute of Cognitive Neurology)^2^. Three initial volumes of each functional run were discarded. Image preprocessing included realignment, field-map-based correction of susceptibility-induced geometric distortions (“Realign and Unwarp”) (Andersson et al., 2001), coregistration of functional and structural images; segmentation of the structural images; spatial normalization to the Montreal Neurological Institute template; and spatial smoothing with a Gaussian kernel of 8 mm full width half maximum. After the realignment, we examined the head motion parameters and discarded each run that showed maximal volume-to-volume (framewise) displacement along any axis exceeding the size of a voxel. Given the nature of the task, we suspected substantial correlations between task and head motion. Therefore, the ICA strategy was used for head motion correction to address this problem as well as to unify the data preprocessing for both primary activation and follow-up task-based connectivity analyses. The ICA analysis is applicable to sparse sampling as well as to continuous fMRI data (Yakunina et al., 2016). The motion-induced components were removed from the spatially smoothed time-series with ICA-AROMA software for both spatially smoothed and unsmoothed data (Pruim et al., 2015).

After the preprocessing, a general linear model (GLM) was used to reveal voxel-wise effects of the experimental conditions on the BOLD signal change (activation). Several periods within each trial, namely the initial period (before the red frame), the main silent “red-frame” period, the final period of problem-solving, the response, and the feedback were modeled according to their actual duration (see the experimental trial outline, Figure 2), with the exception of the main “red-frame” silent period, which was ascribed a 4-second earlier onset in order to fully capture and incorporate the BOLD signal represented by the two volumes minimally contaminated by the head motion. The five periods were modeled separately for the group and individual conditions. Among the five periods, only the main silent period was further used as a condition of interest, as the one backed up by the most careful experimental control in terms of head motion and the amount of overt speech; the other periods were included in the model for the sake of precision. The audio control condition as well as the rest (fixation) condition were explicitly included in the model. The canonical hemodynamic response function without time derivatives was used as the basis function. Because the effects of head motion and motion-by-susceptibility interaction were mainly accounted for at the “Realign and Unwarp” and ICA-AROMA preprocessing steps, head motion parameters were not included in the model.

A one-sided *t*-contrast of the effect of the silent “red-frame” period in the collective vs. individual conditions was computed for each single subject. To control for activations produced by listening to the speech of the other participants, this contrast was masked with an “audio condition vs. rest” contrast; all voxels significant for the latter contrast at *p* < 0.05 were excluded from analysis. Due to the spatial variability of the exclusion mask, the coverage of the resulting contrast image varied across subjects so that data in some within-brain voxels were missing in a certain percentage of participants. Therefore only voxels with data present for at least 50% of the subjects were analyzed. The group-level random-effect analysis with treatment of missing data and removal of outliers was performed with GLMFlex2 (A. P. Schultz)^3^. Activation maps were thresholded with false discovery rate (FDR) correction for multiple comparisons at the voxel level (*p* < 0.05, *q* < 0.05) and an arbitrary extent threshold of 10 voxels (640 mm^3^). Anatomical brain regions were identified on the basis of the Harvard-Oxford probabilistic brain atlas (Desikan et al., 2006) and manually verified by the averaged spatially normalized anatomical images of all subjects.

Functional connectivity analysis was performed with the CONN functional connectivity Toolbox Version 17a (Whitfield-Gabrieli and Nieto-Castanon, 2012)^4^. Initial data preprocessing and condition specifications were the same as for the activation pipeline. Additionally, before application of the ICA-AROMA correction step, head motion artifact and signal spike detection was performed with the Artifact Detection Toolbox (ART)^5^ with liberal threshold settings. Subjects for whom the portion of the outlier scans identified by ART exceeded 0.2 were excluded from the further connectivity analysis. The outliers were subsequently included as nuisance regressors into the denoising linear model along with the linear detrending term. The main BOLD-signal effects of all conditions were regressed out to restrict the analysis to within-condition connectivity alterations rather than global changes of the correlation evoked by the task onset or offset.

For the ROI-based connectivity analysis, the high-pass filter was applied with a cutoff of 0.0025 Hz (1/2D, where D is the duration of the experimental condition cycle). The signal from each ROI was extracted only from gray matter voxels of the unsmoothed functional volumes, to avoid any additional risk of contaminating the data with white matter, CSF, or other ROI signals. The first ROI list included clusters obtained in the “group vs. individual” contrast of the group-level activation analysis. Secondly, we conducted a so-called functional network connectivity (FNC) analysis, and employed ROIs for the eight standard networks from the Conn Toolbox Networks Atlas identified on the basis of the Human Connectome Project. To perform the FNC analysis, all regions within each network were aggregated to form one ROI per network. For the second (group) level connectivity analysis, two-sided “group silent vs. alone silent” *t*-contrasts were specified, and the mean-centered portion of the outlier scans was included as a subject-specific covariate. An “audio control vs. fixation” contrast as well as contrasts between the problem solving and control conditions were additionally examined to facilitate inference. For comparisons including the audio control condition the mean-centered difference in framewise displacement between the contrasted conditions was included into the model (see the Results section for details of the potential head motion confounds analysis).

To derive the spatial map for the default mode network (Raichle et al., 2001; Raichle, 2010) from our own data, we adopted a model-free voxel-to-voxel approach (Yakunina et al., 2016 for applicability of this type of analysis to sparse sampling fMRI data). We used the method of Fair et al. (2007) to obtain the intrinsic connectivity (resting-state like) networks from ICA analysis on the residual time-courses from the entire session data. The smoothed functional data were used, and a standard bandpass filter of 0.008–0.09 Hz was applied.

Due to the large variance in the definition of the default mode network (DMN), overlap of the social brain with the DMN was tested by several methods on the basis of (a) templates from the study by Schultz et al. (2014) thresholded at the value of 100; (b) DMN maps obtained from our data by Fair et al. (2007) method; and (c) seeds from the social-affective default network atlas (Amft et al., 2015) and social brain connectome atlas (Alcala-Lopez et al., 2017).

### Control Experiment

As natural discussion is of greater interest than scrambled utterances, any specific neural correlates of group activity compared to individual activity revealed in the main experiment could represent the neural correlates of the participant’s attention to the speech of the others or the semantic processing involved in discourse comprehension. A control study was run to assess the effects of speech comprehension and attention toward speech and to differentiate these effects from other mental processes involved in cooperative social interaction. A separate group of 24 participants was recruited from Moscow’s academic community (native speakers of Russian, mean age 25 ± 7 years, 16 women). In the scanner, they listened to 30-second passages from two audio books, a detective story read by a female voice and an anti-utopia story read by a male voice, which alternated with scrambled stimuli. The scrambled stimuli were created from the same audio book excerpts in the same way as the stimuli for the audio control condition of the main experiment. Original and scrambled passages were interspersed with 15-second resting baseline periods without any auditory stimulation other than the scanner acoustic noise. Imaging protocols were the same as in the main experiment with the exception of the number and duration of functional sessions. Four 13-minute sessions were completed per subject (two runs per book), and 101 functional volumes were collected per session. Data preprocessing and modeling also replicated those of the main experiment. Voxels showing the effect of the scrambled stimuli compared to the resting baseline at the voxel-wise statistical threshold *p* < 0.05 (uncorrected) were masked out, and group-level activation maps for the contrast of listening to the actual vs. scrambled discourse were statistically thresholded at the same level as the results of the main experiment [FDR correction for multiple comparisons at the voxel level (*p* < 0.05, *q* < 0.05) and an arbitrary extent threshold of 10 voxels (640 mm^3^)] and further compared to the main results. Connectivity effects were examined with a ROI-to-ROI approach for the same two set of ROIs as in the main experiment: the potential social brain components derived from the main experiment data and the major eight intrinsic connectivity networks (FNC analysis). The “actual vs. scrambled discourse” and “scrambled speech vs. baseline” contrasts were assessed in each ROI-to-ROI analysis. The mean-centered portion of the outlier scans was used as a subject-specific covariate.

## Results

### Behavioral Data

Trials for which no response was registered for any reason were excluded from scoring. Data were collapsed across the A and B task subsets. The average accuracy was 73.7% correct (*SD* = 8.6%) in the group condition and 56.3% (*SD* = 13.3%) in the individual condition. A paired sample *t*-test was performed to test for a significant difference [*t*(23) = 6.52, *p* < 0.001]. Only one participant out of 24 demonstrated higher performance in the individual condition.

The significantly higher performance observed in group versus individual problem-solving conditions cannot be considered evidence of a group superiority effect or greater difficulty of the individual condition. Direct comparison of n groups of m members (n × m individuals) with n individuals favors groups for purely statistical reasons; in problems with one objectively correct answer (accuracy tasks) (Steiner, 1966) the likelihood of finding a solution is higher with three people than only one person. Thus, the optimal method of comparison implies a contrast of interacting groups with aggregates of the same number of non-interacting individuals (Laughlin et al., 2006; Pavitt, 1998). However, in our study, the responses of the second and third players were not collected in the individual performance condition because such a procedure may have elicited competition within the team, as seen in some pilot groups. Therefore, we used a more conservative baseline calculated according to the model of Lorge and Solomon (1955): *S* = 1-*F*^*m*^, where *S* is the likelihood that the group solves the problem, *F* is the likelihood that each team member fails to solve the problem, and *m* is the number of team members. Taking the average frequency of errors made by Player 1 in the individual condition as an estimate of *F*, we found that teams may be considered as accurate as individuals if they give 91.6% correct responses. However, the teams significantly underperformed these baselines [*t*(23) = −10.48, *p* < 0.001], demonstrating a group process loss rather than any gain.

### Functional Imaging Data

Data from one participant in the main experiment and one participant in the control experiment were excluded from all neuroimaging statistical analyses due to excessive head motion (more than voxel size along any axis in every run). Four individual runs from three other participants in the main experiment were also discarded for the same head motion criteria or technical reasons.

For the remaining dataset in the main experiment, the mean scan-to-scan (framewise) displacement (*n* = 23) was 0.45 ± 0.55 and 0.45 ± 0.56 mm for the individual and group problem solving conditions, respectively (silent red-frame part of the trial); 0.49 ± 0.67 mm for the resting condition and 0.35 ± 0.53 mm for the scrambled audio control condition. Significant between-condition differences in terms of head motion were only found for the scrambled audio control condition [vs. silent group problem solving: *t*(22) = −2.77, *p* = 0.011 and vs. resting: *t*(22) = −3.77, *p* = 0.001]. Therefore, due to the special sensitivity of the connectivity analysis to head motion, for the FNC comparisons including the scrambled audio control condition, a covariate representing the mean-centered difference in framewise displacement between the contrasted conditions was included into the model. In the control experiment, the mean framewise displacement (*n* = 23) was 0.16 ± 0.09 mm at rest and in the scrambled audio condition; 0.15 ± 0.08 mm in the normal discourse comprehension (audiobook) condition. No between-condition differences in head motion were significant.

The median (semi-IQR) percentage of outlier scans identified by ART (head motion global signal intensity outliers) was 0% (4%) and 0% (2%) for the individual and group problem solving conditions, respectively (silent red-frame part of the trial); 2% (5%) for the resting condition and 2% (3%) for the scrambled audio control condition; 0% (0%) for all conditions of the control experiment. There were no significant between-condition differences in terms of the percent of outliers (two-sided paired Wilcoxon rank sum test), so it was not a condition-specific but overall mean-centered portion of outlier scans per subject that was included as a covariate in all models.

**FIGURE 4:**
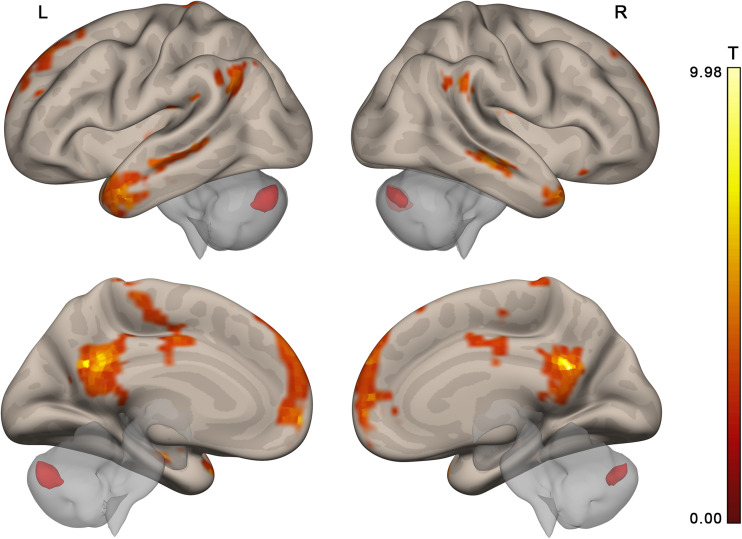
Brain activation specific to collective compared to individual problem solving (contrast “collective silent period >individual silent period,” group random effect analysis, *n* = 23, voxel-wise FDR-corrected level *p* < 0.05, *q* < 0.05, extent threshold *k* = 10) overlaid upon the MNI-space brain template. Voxels activated by scrambled speech vs. rest were excluded from the analysis at the single subject level with a threshold of *p* < 0.05 (uncorrected).

**FIGURE 5:**
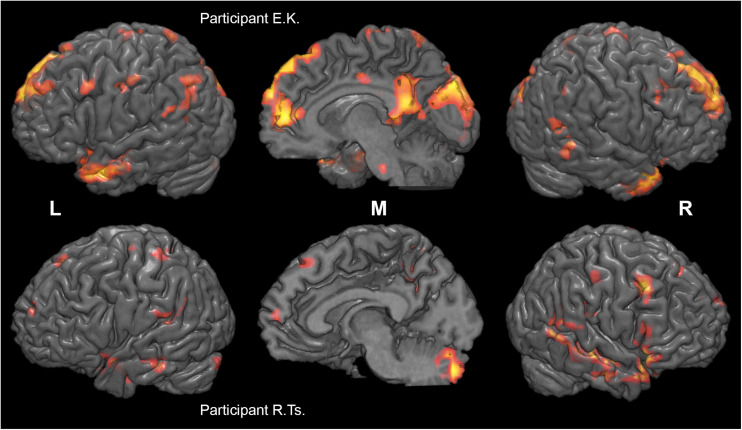
Individual differences in neural substrates specific to collective compared to individual problem solving. Activation maps from the two sample individual participants (E. K., top, and R. Ts., bottom) are thresholded at *p* < 0.005 (uncorrected), *k* > 10, and rendered on individual anatomical images using MRICroGL (C. Rorden, http://www.mccauslandcenter.sc.edu/mricrogl/). Voxels activated by scrambled speech vs. rest at *p* < 0.05 (uncorrected) are masked out.

Two more participants were further excluded from the connectivity analysis in the main experiment and one in the control experiment. In these participants, the percentage of scans invalidated due to head motion or signal spikes before denoising exceeded 20%. This resulted in *n* = 23 and *n* = 24 for the activation analysis, *n* = 21 and *n* = 23 for the connectivity analysis, main and control experiments, respectively. The contrast of collective and individual conditions revealed activation in multiple brain areas (see Figure 4 and Tables 1, 2), including several subdivisions of the prefrontal cortex (dorsomedial: dmPFC, ventromedial: vmPFC, and dorsolateral: dlPFC), cingulate cortex (anterior: ACC, posterior: PCC), retrosplenial cortex, precuneus, bilateral frontal and temporal poles, lateral parietal cortex bilaterally (LLP and RLP), basal and middle temporal areas, insula and operculum, primary motor and somatosensory cortices, supplementary motor area (SMA), and the cerebellum. Substantial individual differences in the activation pattern are illustrated in Figure 5. The whole-brain analysis revealed no significant correlations between the main contrast activation and behavioral covariate, reflecting the difference between group and individual performances (percent of group minus percent of individual correct responses).

**TABLE 1:** Regions of greater activation in group compared to individual problem solving: Overlap with the DMN, control experiment activation data, and social brain atlas.

			Overlap with DMN, %			
	Anatomic	Cluster			Overlap with Control	Social Brain Regions
Cluster #	Description	Size, vx	ICA	Template	Experiment Results, %	(Alcala-Lopez et al., 2017)
1	Medial Prefrontal Cortex and Frontal Pole (bilateral)	331	72.5	93.3	22.1	dmPFC, frontal pole
2	Posterior Cingulate Cortex, Retrosplenial Cortex, and Precuneus (bilateral)	191	94.8	77.9	2.6	PCC
3	Temporal Pole, Superior Temporal Gyrus, and Middle Temporal Gyrus (left)	98	19.4	46.3	35.7	lMTG, l temporal pole
4	Cerebellum, Crus (left)	94	27.7	26.7	0.0	none
5	Temporal Cortex and Insula (left)	88	20.5	34.0	20.5	none
6	Anterior Cingulate Cortex and Precentral Gyrus (left)	81	55.6	0.0	0.0	none
7	Angular Gyrus (left)	77	100.0	93.8	54.6	lTPJ
8	Cerebellum, Crus (right)	70	0.0	67.9	67.1	none
9	Temporal Pole (right)	39	23.1	51.0	46.2	r temporal pole
10	Operculum (left)	37	24.3	0.0	2.7	none
11	Temporal Cortex (right)	36	33.3	50.0	0.0	none
12	Angular Gyrus (right)	21	100.0	22.0	0.0	none
13	Postcentral Gyrus (bilateral)	20	5.0	0.0	0.0	none
14	Insular Cortex and Parietal Operculum (right)	15	0.0	0.0	0.0	none
15	Superior Frontal Gyrus (right) and Supplementary Motor Area (left)	10	0.0	21.3	0.0	none

*Cluster sizes are given in voxels (4 × 4 × 4 mm). Overlap with DMN column: the percent of the cluster volume covered by DMN map obtained from the same data with ICA and from DMN template (Schultz et al., 2014). Overlap with Control Experiment Results column: the percent of cluster volume covered by the activation map for discourse comprehension in the control experiment. “Social brain” column shows whether the cluster includes center of mass for seeds from Alcala-Lopez et al. (2017) social brain connectome atlas.*

**TABLE 2:** Regions of greater activation in group compared to individual problem solving: Peak coordinates and anatomic labels.

			LH				RH		
Cluster #	Region Label	*t*	MNI Coordinates			*t*	MNI Coordinates		
			x	y	z		x	y	z
1	Paracingulate Gyrus	5.96	−8	54	0	4.87	10	52	10
1	Frontal Pole	4.77	−20	42	20	5.38	10	56	30
1	Frontal Medial Cortex					4.73	2	52	−14
1	Superior Frontal Gyrus	4.39	−2	52	42				
1	Cingulate Gyrus, ant.					3.09	10	36	−2
2	Cingulate Gyrus, post.	6.70	−10	−48	30	9.98	6	−52	26
2	Precuneus Cortex	7.44	−10	−56	26				
3	Temporal Pole	7.64	−48	2	−32				
3	Middle Temporal Gyrus, post.	3.74	−54	−12	−26				
3	Superior Temporal Gyrus, post.	3.43	−46	−4	−22				
4	Cerebellum, Crus I*	7.24	−26	−84	−34				
4	Cerebellum, Crus II*	3.97	−10	−92	−30				
5	Planum Temporale	5.77	−38	−36	2				
5	Insular Cortex	5.23	−34	−28	2				
5	Middle Temporal Gyrus, post.	5.06	−44	−22	−12				
5	Parahippocampal Gyrus, ant.	5.01	−34	−16	−22				
5	Temporal Fusiform Cortex, post.	3.55	−42	−28	−18				
6	Cingulate Gyrus, ant.	4.40	−6	−8	42				
6	Precentral Gyrus	4.01	−4	−26	60				
7	Angular Gyrus	6.90	−62	−60	26				
8	Cerebellum, Crus II*					6.47	26	−84	−38
8	Cerebellum, Crus I*					4.94	34	−76	−34
9	Temporal Pole					6.79	50	16	−30
9	Frontal Orbital Cortex					3.42	34	20	−18
10	Parietal Opercular Cortex	5.35	−30	−32	22				
10	Central Opercular Cortex	3.21	−42	−16	18				
11	Middle Temporal Gyrus, post.					5.39	66	−24	−10
11	Temporal Fusiform Cortex, post.					4.20	42	−32	−14
11	Planum Polare					3.25	42	−16	−14
12	Angular Gyrus					5.20	58	−48	26
13	Postcentral Gyrus	4.20	−6	−36	78	4.36	6	−36	66
14	Insular Cortex					3.83	30	−24	18
14	Parietal Opercular Cortex					3.80	38	−24	30
15	Superior Frontal Gyrus					3.26	18	32	62
15	Supplementary Motor Area*	4.35	−2	24	62				

*Label obtained from AAL or cerebellum (flirt) atlases and manual inspection. One peak with the maximal t-value per cluster and region is presented. LH — left hemisphere; RH — right hemisphere.

Given the resemblance of the list of brain regions activated by group problem solving to those of the DMN, the activation maps and DMN were compared. The thresholded DMN template from Schultz et al. (2014) covered 60% of the activation volume, and the ICA-based DMN map obtained from our data by Fair et al. (2007) method covered 55% of the activation volume. The overlap of each activation cluster with the DMN is shown in Table 1. The last column of Table 1 indicates if the center of mass of any seed regions from the social brain atlas by Alcala-Lopez et al. (2017) falls within the scope of activation clusters obtained in our study.

**FIGURE 6:**
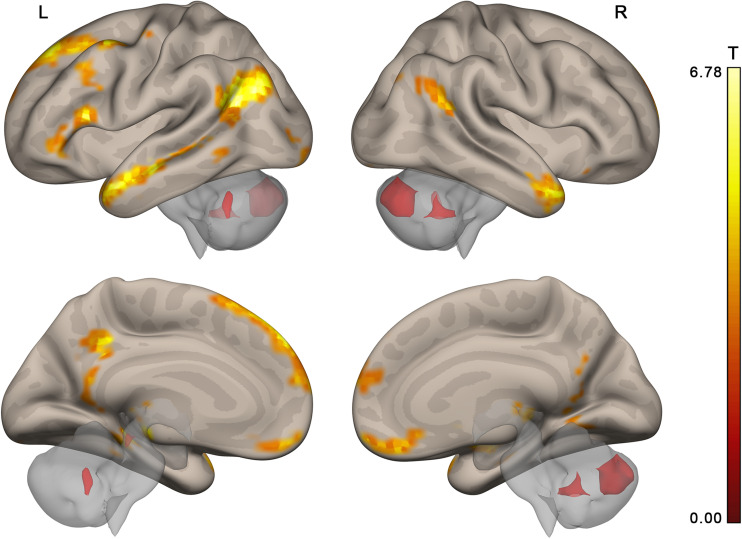
Brain activation specific to discourse comprehension compared to individual word comprehension revealed in a control experiment (contrast “actual discourse >scrambled discourse,” group random effect analysis, *N* = 24, voxel-wise FDR-corrected level *p* < 0.05, *q* < 0.05, extent threshold *k* = 10) overlaid over the MNI-space brain template. Voxels activated by scrambled speech vs. resting baseline were excluded from the analysis at the single subject level with a threshold of *p* < 0.05 (uncorrected).

**FIGURE 7:**
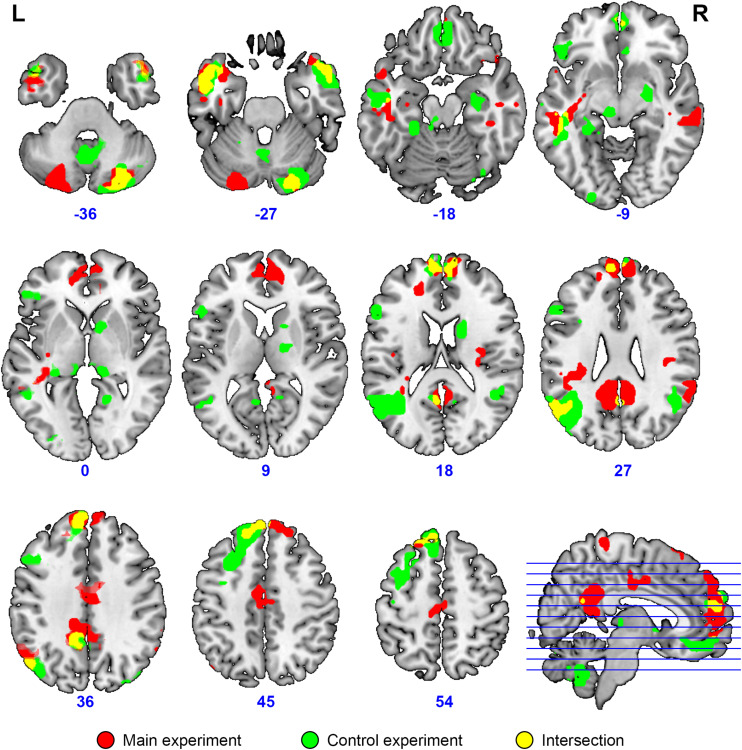
Overlap (yellow) of brain activation specific to collective compared to individual problem solving (main experiment, red, *n* = 23) and specific to normal discourse comprehension compared to listening to scrambled speech (control experiment, green, *N* = 24). Activation was significant at the voxel-wise FDR-corrected level *p* < 0.05, *q* < 0.05, extent threshold *k* = 10. The image is overlaid on top of the mean MNI-space normalized anatomical images of the participants from the main experiment. Voxels activated by the scrambled speech vs. rest at the uncorrected level of *p* < 0.05 were excluded from the analysis at the single subject level in both experiments.

The activation location revealed in the “collective vs. individual” contrast was also compared to the activation location for listening to meaningful vs. scrambled audio book excerpts in the control study. The activation revealed for this contrast in the control experiment is presented in Figure 6, Table 3, and Supplementary Table S1; 51% of its volume overlapped with the DMN map derived from the data of the main experiment; also 51% (similar but not identical set of voxels) overlapped with the thresholded DMN template. Overall, 20% of the activation volume from the main experiment overlapped with the results of the control experiment. Partial overlaps are observed for the activation clusters in the temporal poles, mPFC, PCC, LLP, and right cerebellum (see Figure 7 and Table 1). Remarkably, while activation in the RLP was observed in both studies, the activation sites did not overlap.

**TABLE 3:** Regions of greater activation evoked by meaningful versus scrambled audiobook in the control experiment.

			Highest Peak				
Cluster #	Anatomic Description	Cluster Size	*t*	Side	MNI Coordinates		
					x	y	z
1	Frontal Pole (bilateral), Middle Frontal Gyrus (left), Superior Frontal Gyrus (left), Precentral Gyrus (left), Paracingulate Gyrus (right)	315	6.50	L	−6	52	42
2	Angular Gyrus (left), Lateral Occipital Cortex (left)	210	6.79	L	−40	−58	20
3	Cerebellum* (right)	147	5.46	R	18	−88	−34
4	Temporal Pole (left), Middle Temporal Gyrus (left)	132	5.90	L	−50	4	−26
5	Cerebellum* (bilateral)	90	6.26	L	−2	−60	−46
6	Inferior Frontal Gyrus (left), Middle Frontal Gyrus (left), Frontal Orbital Cortex (left)	87	5.93	L	−54	20	14
7	Middle Temporal Gyrus (right), Temporal Pole (right), Frontal Orbital Cortex (right)	72	5.86	R	50	4	−30
8	Frontal Pole (left), Frontal Medial Cortex (right)	62	5.20	L	−2	56	−10
9	Cingulate Gyrus, post. (left), Precuneus Cortex (bilateral)	62	5.01	L	−10	−44	38
10	Parahippocampal Gyrus (left), Temporal Fusiform Cortex (left)	41	5.44	L	−26	−36	−10
11	Subcallosal Cortex, (right)	39	5.34	R	10	8	−2
12	Angular Gyrus (right)	33	5.47	R	46	−44	22
13	Parahippocampal Gyrus, ant. (right)	29	4.28	R	22	−4	−10
14	Parahippocampal Gyrus, post. (left)	22	4.91	L	−6	−24	−10
15	Cingulate Gyrus, post. (right), Lateral Occipital Cortex, sup. (left), Occipital Pole (left)	16	4.51	R	14	−32	2
16	Lateral Occipital Cortex, sup. (right)	11	3.84	R	34	−84	34
17	Lingual Gyrus (right)	10	3.71	R	16	−54	0

*Label obtained from AAL or cerebellum (flirt) atlases and manual inspection. Cluster sizes are given in voxels (4 × 4 × 4 mm). One peak with the maximal t-value per cluster and region is presented.

Fifteen clusters obtained from the contrast of the group vs. individual silent conditions in the activation analysis were used as ROIs in a follow-up functional connectivity analysis. Within that specified ROI set, no significant (FDR-corrected) ROI-to-ROI functional connectivity modifications were revealed in a contrast for group vs. individual problem solving (silent conditions). The FNC analysis tested modifications of between-network functional connectivity for eight major networks (DMN, FPCN, sensorimotor, language, dorsal attention, visual, salience and cerebellar) with a two-sided *t*-contrast with a FDR correction for multiple comparisons for individual connections at the analysis level (*p* < 0.05, *q* < 0.05). The results revealed that the group problem solving condition is characterized by decreased connectivity between the language and salience networks compared to the individual condition [*t*(19) = −4.56, *p*(FDRcorr) = 0.006]. Interestingly, there was a significant decrease in connectivity between multiple pairs of functional networks during the scrambled audio control condition compared to the fixation control as well as both problem solving conditions (see Supplementary Figure S1). At the same time, the fixation condition significantly differed from the group problem solving by only one connection (decreased synchronization between the sensorimotor and dorsal attention networks, [*t*(19) = −3.63, *p*(FDRcorr) = 0.0495], and from the individual problem solving by two connections of the sensorimotor network [the same desynchronization with the dorsal attention network, *t*(19) = −4.98, *p*(FDRcorr) = 0.0002, and increased coupling with the language network, *t*(19) = 3.54, *p*(FDRcorr) = 0.008]. No significant connectivity effects were observed in a control experiment contrasting normal vs. scrambled discourse comprehension and scrambled discourse vs. resting control conditions.

## Discussion

In this study we aimed to explore the neural basis of real group problem solving using a paradigm providing ecologically valid settings for fMRI. We used behavioral, activation and functional connectivity data to address the question of whether a group is more than the sum of its members at the level of brain activity. Although at the behavioral level a group superiority effect may be expected in long-existing groups, our participants demonstrated a significant group loss in their task performance. In contrast, our neuroimaging results provided evidence that, in terms of brain activation, a group is more than the sum of its members. A number of brain areas including the medial prefrontal cortex, the lateral parietal cortex, the cingulate cortex, and the precuneus as well as the frontal and temporal poles manifested greater activation during group vs. individual problem solving. However, the connectivity among these areas did not show significant increases, thus suggesting that the potential components of the social brain are co-activated rather than form a holistic network during real cooperative activity. Instead, with the FNC analysis examining task-based connections between the major intrinsic connectivity networks we discovered decreasing connectivity between the language and the salience networks in the group vs. individual activity conditions. Therefore, we propose that the social mode of the brain may be described as a re-configuration of connectivity between basic networks, rather than the actualization of a specific social brain network(s).

Most of the areas that were significantly more activated during the group vs. individual problem solving in our experiment are on the list of brain regions considered to be components of the social brain (Kennedy and Adolphs, 2012) including the mPFC, which was also activated in both previous fMRI studies that directly compared cooperative interactions to individual task accomplishment (Decety et al., 2004; Sebanz et al., 2007). Other areas located in coordinates reported by Decety et al. (2004) were not implicated. Therefore, our results only partially replicated two previous fMRI studies which involved both online interactions (simulated in one study and real in the other) and comparisons of group and individual activity. Possible reasons for such a discrepancy are the small number of participants in the previous studies, high inter-individual variability, or methodological differences. The latter include the mode of social interaction [alternating cooperation and competition tasks in the procedure used by Decety et al. (2004), or the entirely cooperative setting in our study and in that by Sebanz et al. (2007)], differences between simulated and real interactions, and task differences.

The social brain has recently been reconsidered; rather than consisting of a set of individual components, it is thought to be structured as a set of large-scale networks, with the mirror neuron system (MNS) and mentalizing system (MENT) being the most important (Frith and Frith, 2010; Kennedy and Adolphs, 2012; Schilbach et al., 2013; Redcay and Schilbach, 2019). Within this framework, we can describe our findings primarily as activation within the MENT system (dmPFC, temporal poles, PCC and retrosplenial cortex), with some activation in the motor network that can be considered part of the MNS. However, for only 7 out of 36 seed regions included in the social brain atlas by Alcala-Lopez et al. (2017) on the basis of the co-activation and resting state connectivity analysis, the center of mass was within the clusters of activation revealed during the real cooperative activity in our study (see Table 1, last column).

In terms of Alcala-Lopez et al. (2017) the social brain areas identified during real group problem solving belong mostly to the set of “higher-order” (associative) seeds that includes the dmPFC, frontal pole, PCC and precuneus, TPJ, middle temporal gyri and temporal poles, while the cerebellum and SMA are rather classified as “intermediate level.” At the same time, we registered almost no involvement of areas from the visual-sensory and limbic sets. The fact that in the group reasoning condition we did not find increased activation in areas involved in face processing, biological motion processing and joint attention, such as the fusiform face area (FFA), superior temporal sulcus (STS) or orbitofrontal cortex, may be due to the lack of face-to-face visual contact between Player 1 and the other two players, or to the exclusive masking of all areas responding to sound (e.g., STS).

Notably, about two thirds of the brain volume activated in group problem solving versus individual problem solving includes structures of the DMN. One possible explanation is that the DMN is usually less activated during goal-directed activity than rest, and for two goal-directed tasks the greater DMN activation may be observed for the task with fewer demands on executive functions (Spreng, 2012). Taking this into account, greater partial activation of the DMN in group problem solving compared to individual problem solving may reflect less difficulty of the group process, or at least fewer executive function requirements. This view is supported by an observation that “activation” of the mPFC by many social cognition fMRI tasks compared to control tasks is a result of lesser deactivation rather than greater increment of the BOLD signal compared to baseline (Iacoboni et al., 2004; Schilbach et al., 2008). This pattern is mostly reproduced in our real cooperative interaction data. Nonetheless, the way to directly measure and compare the subjective difficulty of solving a task in a collective discussion and individually is not clear.

Some indirect evidence is provided by the fact that individual differences in the specific neural correlates of the group vs. individual activity showed no correlation with the behavioral indicator of the difference between the participant’s performance in the group and individual conditions. Therefore, we should consider these differences as potential variations in the functional system of the social brain or in the “style” of the social brain performance rather than the greater or lesser involvement of the social brain in different participants resulting in their respective higher or lower performance in collective tasks.

On the other hand, the DMN and frontoparietal (executive) network have been shown to be co-activated rather than anti-correlated for some tasks involving creative idea generation, imagination and social working memory (Spreng, 2012). Overlap of the social brain, mainly the MENT network, and the DMN has repeatedly been reported in earlier “offline” studies (Schilbach et al., 2008; Mars et al., 2012) as well as in a conjunction meta-analysis (Schilbach et al., 2012) suggesting a similarity of the two systems’ functions. This idea is supported by the vast literature discussing mentalization processes (namely theory of mind, perspective-taking and introspection abilities) as possible candidates for the “common denominators” of the two networks (Andrews-Hanna et al., 2010; Kennedy and Adolphs, 2012; Mars et al., 2012; Schilbach et al., 2012). Schilbach et al. (2012) and later Amft et al. (2015) conceptualized the MENT and DMN as the “socio-affective default” (SAD) system, two key hubs of which (dmPFC and precuneus) also fell within the scope of our results.

Our results reveal the neural substrate of both reasoning and communication, and it seems impossible to empirically disentangle these two components of group problem solving. A separate control experiment was conducted to rule out the possibility that the greater activation observed in the group condition is evoked exclusively by communicative processes – specifically, an understanding of the utterances of the other players and paying attention to their discourse – as the effects of voice and typical lexical units were already excluded in the main experiment. The control experiment contrasted brain activation while listening to regular and scrambled audio books. The intersections of the activation maps from our two experiments within the MENT system areas (mPFC, PCC, temporal poles) are not surprising if we consider mentalization to be intrinsically involved in any form of discourse comprehension, regardless of whether we discuss a problem or listen to a fiction book. The same contrast implemented for reading revealed similar activation in the left temporal pole as in an earlier study (Vandenberghe et al., 2002). A partial overlap of the neural correlates of active comprehension of meaningful discourse and the DMN also suggests that coincidence of the activation in the group reasoning condition and the DMN should be explained by the similar processes underlying the DMN and cooperative interaction, rather than by greater difficulty or more pronounced cognitive control in the individual condition.

Interestingly, the neural correlates of both group problem solving and audiobook comprehension demonstrated left-sided asymmetry. This observation is in accordance with the data collected by Alcala-Lopez et al. (2017) although Hyatt et al. (2015) have shown that among DMN subnetworks the one associated with the right, but not left, TPJ is recruited by mentalizing more than by mere semantic tasks. Evidence for lateralization of the increased brain-to-brain synchrony in the TPJ that comes from fNIRS hyperscanning research is also mixed and varies across tasks and designs (Wang et al., 2018 for a review).

Until recently, the components of the social brain were mainly identified in offline laboratory studies lacking ecological validity and focused on the study of the function of regions taken in isolation. Similarly, connectivity within the social brain was mainly deduced from co-activation statistics (Amft et al., 2015; Alcala-Lopez et al., 2017). However, co-activation does not necessarily imply connection. Given the data from the social brain at work, we addressed the question whether the social brain areas identified in group vs. individual problem solving would also behave as a holistic network, demonstrating modified connectivity patterns during the cooperative activity. The task-based functional connectivity analysis has shown that this is not the case, although the collected data were sufficient to identify major networks in ICA and ROI-to-ROI analyses.

Given this evidence we proposed that the social mode of the brain may be described as a re-configuration of connectivity between basic networks, rather than functioning of a specific social brain network. The DMN and sensorimotor networks (as representatives for MENT and MNS, respectively) were considered as primary candidates for such modified connectivity. However, in the group compared to the individual condition, we found decreased connectivity between two other networks, namely the language and salience networks, which may reflect some changes in the interplay between speech, attention, and thought in different modes of reasoning. Since a similar connectivity decrease is observed in the scrambled audio control condition compared to the fixation control condition, we cannot completely rule out the possibility that the observed modifications are elicited by speech perception rather than social interaction *per se*. At the same time, between-network functional connectivity did not change in the “audiobook comprehension vs. scrambled speech” contrast of the control experiment, suggesting that the modifications observed in the main experiment cannot be attributed to discourse comprehension.

Interestingly, in the control experiment we did not observe the counterintuitive desynchronization of between-network interactions due to scrambled speech that was seen in the main experiment. Contrarily, at the lenient (uncorrected) statistical threshold we found increased coupling of the dorsal attention network with several other networks (the DMN, language, and salience networks), as would be expected. So, the scattered speech synchronizes the networks in the control experiment but decouples them in the main experiment. A plausible interpretation for this fact, supported by remarks from some participants of the main experiment, is that the fixation might have acted as a third problem solving condition rather than rest. Instead of relaxing, at least some of the players might have been trying to solve previously presented problems. That would also explain the coherent difference in major network connectivity between the fixation and each of the two problem-solving conditions (growing synchronization of the sensorimotor and dorsal attention networks). Comprising a limitation of the present study, the problem of the resting control condition calls for further improvement of our new methodology.

Overall our FNC results demonstrate that functional connectivity analysis of the task-based fMRI data obtained from a real complex cooperative task is a promising and productive approach to understanding the social brain at work.

## Conclusion

The present study revealed specific neural substrates of complex cooperative interactions, in this case group versus individual problem solving, illustrating that a group is more than the sum of its members in terms of brain activation. The revealed substrates include the mPFC, frontal and temporal poles, and cingulate and retrosplenial cortices as well as precuneus. These areas were previously identified as potential components of the so-called social brain on the basis of offline judgments of material related to socializing. Thus, our results provide evidence for the actual involvement of these components in real-time social interactions. However, the connectivity analysis revealed that the co-activated social brain components did not increase their coupling during cooperation, as would be expected for a holistic network. Instead, we suggest that the social mode of the brain may be described as a re-configuration of connectivity between basic networks, and we found decreased connectivity between the language and salience networks in the group compared to the individual condition. While the present data are not sufficient to completely delineate activation specific to collective reasoning from that underlying communication within a group, we believe that the developed paradigm for neuroimaging of individuals engaged in complex real group activities will yield future answers to this question and will promote integration of the existing “third-person neuroscience” evidence in the emerging stream of “second-person neuroscience.”

## Data Availability Statement

The dataset sharing requests should be addressed to the corresponding author and need to be approved by the Research Institute of Neuropsychology of Speech and Writing.

## Ethics Statement

The studies involving human participants were reviewed and approved by The Interuniversity Ethics Committee of Moscow. The participants provided their written informed consent to participate in this study. Written informed consent was obtained from the individuals for the publication of any potentially identifiable images or data included in this article.

## Author Contributions

IS conceived the idea. IS and EP designed the study. IS, EP, RV, EM, and VS contributed to the methodology. AR, RR, RV, and EP collected the data. IS, AR, RR, and EP contributed to the data analysis. IS, RV, and EP contributed to the interpretation of results and wrote the initial draft of the manuscript. EM and VS coordinated the study and critically revised the manuscript. All the authors took part in writing, review and editing, and read and approved the current manuscript.

## Conflict of Interest

The authors declare that the research was conducted in the absence of any commercial or financial relationships that could be construed as a potential conflict of interest.

## Footnotes

1 http://iqtest.dk/, v. 3.0.

2 www.fil.ion.ucl.ac.uk

3 http://mrtools.mgh.harvard.edu/

4 http://www.nitrc.org/projects/conn

5 http://www.nitrc.org/projects/artifact_detect
