# Supplementary material for: Neural Correlates of Group Versus Individual Problem Solving Revealed by fMRI
Source: Front Hum Neurosci. 2020 Aug 28;14:290. doi: 10.3389/fnhum.2020.00290 (PMC7483667; doi:10.3389/fnhum.2020.00290)
Supplement: Supplementary file 1 [file Table_1.pdf]

frontiers

Supplementary Material

Supplementary Table 1. Regions of Greater Activation Evoked by Meaningful versus Scrambled Audiobook in the Control Experiment (Extended)

Cluster #	Region	Cluster Size	Peaks				
			t	Side	MNI Coordinates		
					x	y	z
1	Frontal Pole	315	6.50	L	−6	52	42
1	Frontal Pole	—	3.89	R	10	64	18
1	Middle Frontal Gyrus	—	5.43	L	−42	8	58
1	Superior Frontal Gyrus	—	4.73	L	−26	20	50
1	Precentral Gyrus	—	4.73	L	−38	−4	58
1	Paracingulate Gyrus	—	3.43	R	6	52	18
2	Angular Gyrus	210	6.79	L	−40	−58	20
2	Lateral Occipital Cortex, sup.	—	6.59	L	−44	−70	28
3	Cerebellum, Crus I*	147	5.46	R	18	−88	−34
3	Cerebellum, VI*	—	4.77	R	22	−84	−42
4	Temporal Pole	132	5.90	L	−50	4	−26
4	Middle Temporal Gyrus, post.	—	5.81	L	−54	−16	−14
4	Middle Temporal Gyrus, temporoocc.	—	4.49	L	−54	−48	−2

Cluster #	Region	Cluster Size	Peaks				
					MNI Coordinates		
			t	Side	x	y	z
5	Cerebellum, IX*	90	6.26	L	−2	−60	−46
5	Cerebellum, VIIIa*	—	5.00	L	0	−58	−36
5	Cerebellum, IX*	—	4.96	R	14	−48	−46
5	Cerebellum, Vermis IX*	—	4.18	L	−10	−60	−34
5	Cerebellum, Vermis VIIIa*	—	3.92	R	2	−68	−46
5	Cerebellum, Vermis VIIIb*	—	3.88	R	6	−68	−34
6	Inferior Frontal Gyrus, pars operc.	87	5.93	L	−54	20	14
6	Inferior Frontal Gyrus, pars triang.	—	4.56	L	−54	36	2
6	Middle Frontal Gyrus	—	5.46	L	−50	20	38
6	Frontal Orbital Cortex	—	3.99	L	−42	36	−6
7	Middle Temporal Gyrus, ant.	72	5.86	R	50	4	−30
7	Temporal Pole	—	4.11	R	54	16	−26
7	Frontal Orbital Cortex	—	3.21	R	38	24	−22
8	Frontal Pole	62	5.20	L	−2	56	−10
8	Frontal Medial Cortex	—	4.38	R	2	40	−18
8	Subcallosal Cortex	—	3.82	R	6	28	−10

Cluster #	Region	Cluster Size	Peaks				
					MNI Coordinates		
			t	Side	x	y	z
9	Cingulate Gyrus, post.	62	5.01	L	−10	−44	38
9	Precuneous Cortex	—	3.86	R	14	−52	14
9	Precuneous Cortex	—	3.39	L	−6	−56	6
10	Parahippocampal Gyrus, post.	41	5.44	L	−26	−36	−10
10	Temporal Fusiform Cortex, post.	—	3.30	L	−22	−44	−22
11	Subcallosal Cortex	39	5.34	R	10	8	−2
12	Angular Gyrus	33	5.47	R	46	−44	22
13	Parahippocampal Gyrus, ant.	29	4.28	R	22	−4	−10
14	Parahippocampal Gyrus, post.	22	4.91	L	−6	−24	−10
15	Cingulate Gyrus, post.	16	4.51	R	14	−32	2
15	Lateral Occipital Cortex, inf.	—	3.93	L	−34	−88	2
15	Occipital Pole	—	3.63	L	−26	−96	−6
16	Lateral Occipital Cortex, sup.	11	3.84	R	34	−84	34
17	Lingual Gyrus	10	3.71	R	16	−54	0

* Label obtained from AAL or cerebellum (flirt) atlases and manual inspection. Cluster sizes are given in voxels (4×4×4 mm). One peak with the maximal *t*-value per cluster and region is presented.
