# Supplementary material for: Neural Correlates of Group Versus Individual Problem Solving Revealed by fMRI
Source: Front Hum Neurosci. 2020 Aug 28;14:290. doi: 10.3389/fnhum.2020.00290 (PMC7483667; doi:10.3389/fnhum.2020.00290)
Supplement: Supplementary file 2 [file Image_1.pdf]

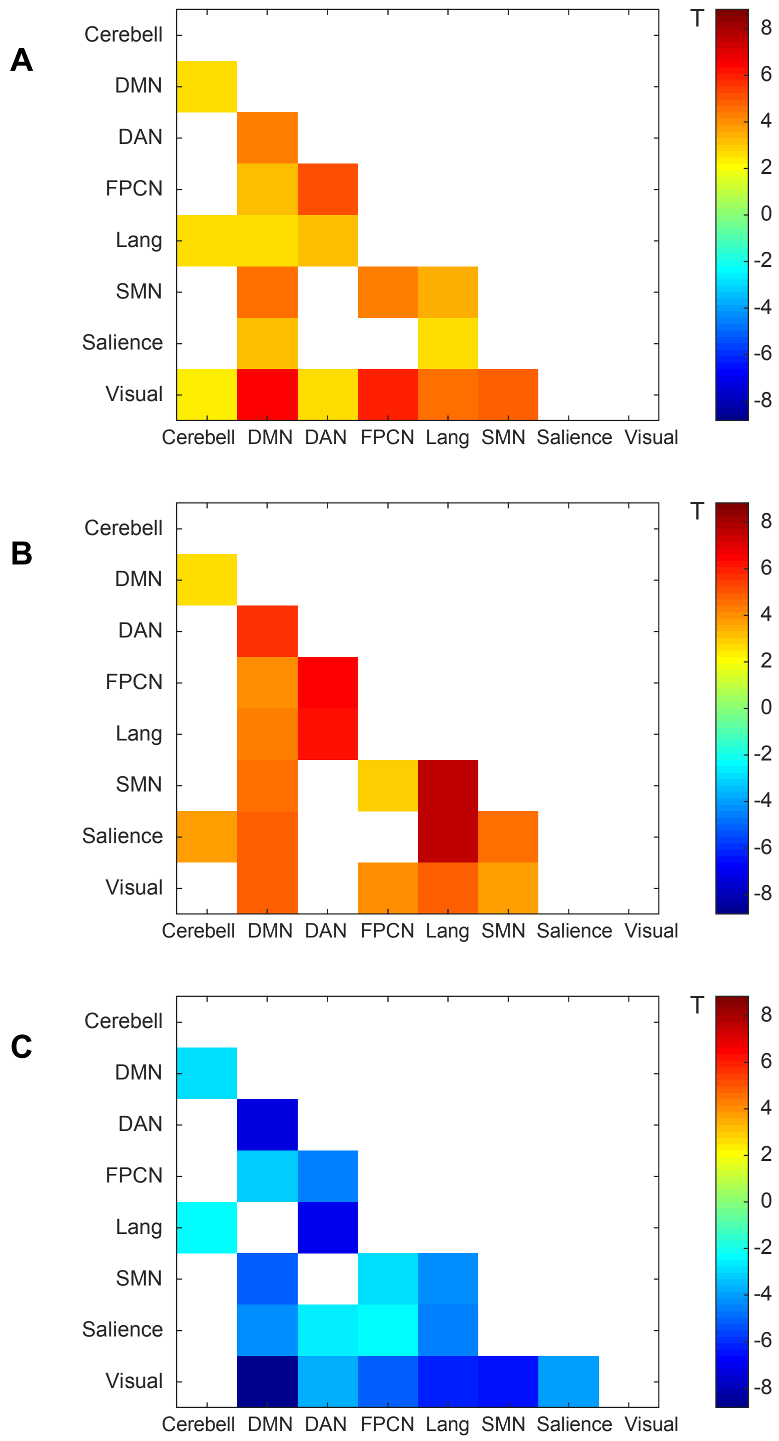


**Supplementary Figure 1.** Significant between-network connections revealed by the FNC analysis in the main experiment. **A:** group problem solving vs. scrambled audio conditions; **B:** individual problem solving vs. scrambled audio conditions; **C:** scrambled audio vs. fixation control conditions. For all contrasts, $n = 21$, statistical threshold: two-sided $t$-test, $p < .05$ (FDR-corrected at the analysis level). Color represents $t$-statistic value. Only significant changes are plotted.
